# Treatment of chronic hemodialysis patients with low-dose fenofibrate effectively reduces plasma lipids and affects plasma redox status

**DOI:** 10.1186/1476-511X-11-47

**Published:** 2012-07-06

**Authors:** Agnieszka Makówka, Przemysław Dryja, Grażyna Chwatko, Edward Bald, Michał Nowicki

**Affiliations:** 1 Department of Nephrology, Hypertension and Kidney Transplantation, Medical University of Łódź, Łódź, Poland; 2 Chair and Department of Environmental Chemistry, University of Łódź, Łódź, Poland; 3 Department of Nephrology, Hypertension and Kidney Transplantation, Medical University of Łódź, University Hospital #1, Kopcińskiego 22, 90-153, Łódź, Poland

**Keywords:** Fenofibrate, Dyslipidemia, Hemodialysis, Oxidative stress, Inflammation

## Abstract

Dyslipidemia is common in chronic hemodialysis patients and its underlying mechanism is complex. Hemodialysis causes an imbalance between antioxidants and production of reactive oxygen species, which induces the oxidative stress and thereby may lead to accelerated atherosclerosis. Statins have been found to be little effective in end-stage kidney disease and other lipid-lowering therapies have been only scarcely studied. The study aimed to assess the effect of low-dose fenofibrate therapy on plasma lipids and redox status in long-term hemodialysis patients with mild hypertriglyceridemia.

Twenty seven chronic hemodialysis patients without any lipid-lowering therapy were included in a double-blind crossover, placebo-controlled study. The patients were randomized into two groups and were given a sequence of either 100 mg of fenofibrate per each hemodialysis day for 4 weeks or placebo with a week-long wash-out period between treatment periods. Plasma lipids, high sensitive C-reactive protein (CRP), urea, creatinine, electrolytes, phosphocreatine kinase (CK), GOT, GPT and plasma thiols (total and free glutathione, homocysteine, cysteine and cysteinylglycine) were measured at baseline and after each of the study periods. Plasma aminothiols were measured by reversed phase HPLC with thiol derivatization with 2-chloro-1-methylquinolinium tetrafluoroborate.

Fenofibrate therapy caused a significant decrease of total serum cholesterol, LDL cholesterol and triglycerides and an increase of HDL cholesterol. The treatment was well tolerated with no side-effects but there was a small but significant increase of CK not exceeding the upper limit of normal range. There were no changes of serum CRP, potassium, urea, and creatinine and liver enzymes during the treatment. Neither total nor total free cysteinylglycine and cysteine changed during the study but both total and free glutathione increased during the therapy with fenofibrate and the same was observed in case of plasma homocysteine.

The study shows that a treatment with reduced fenofibrate dose is safe and effective in reducing serum triglycerides and cholesterol in chronic dialysis patients and may shift plasma aminothiol balance towards a more antioxidative pattern.

## Introduction

The patients with end-stage kidney disease are characterized by accelerated atherosclerosis and greatly increased cardiovascular morbidity and mortality [1,2]. Both conventional and non-conventional cardiovascular risk factors such as lipoprotein (a), homocysteine, inflammation, oxidative stress and a variety of uremic toxins may be involved in the pathogenesis of cardiovascular disease in this population [2,3]. The current unifying pathophysiological concepts of atherosclerosis in subjects with and without renal function impairment emphasize the role of chronic inflammation and oxidative stress in the arterial wall in this process [3,4].

Fibrates have been used for the treatment of lipid disorders for more than 40 years largely due to the effective lowering of serum triglyceride levels. Effects of this class of drugs have been studied in several large clinical trials with cardiovascular end-points including the seminal Fenofibrate Intervention and Event Lowering in Diabetes (FIELD) trial [5]. Despite their well proven renoprotective effect as shown by a reduction of the progression to microalbuminuria in diabetic patients in the Diabetes Atherosclerosis Intervention Study (DAIS) [6] a use of most fibrates including fenofibrate has been limited to patients with normal or only mildly decreased glomerular filtration rate due to the accumulation of their metabolites, increase of serum creatinine and higher risk of rhabdomyolysis [7-9]. As the result the patients with low GFR glomerular filtration rate, i.e. chronic kidney disease stage 3-5day were excluded from all large trials with fibrates precluding an assessment of their efficacy and safety in this clinical setting even with reduced dosing [8,10]. Mixed dyslipidemia associated with chronic kidney disease is characterized by hypertriglyceridemia and low serum HDL-cholesterol [10] that make this population of patients good candidates for fibrate treatment.

The lack of the clinical trial experience with fibrates in the patients with low glomerular filtration rate contrasts both with their renoprotective effects shown in experimental studies [11-13] as well as with everyday practice [14]. The authors of one recent study found that fenofibrate was chronically used by 34 of 305 hemodialysis patients from two centers in Taiwan [14]. On the other hand several series of case reports of rhabdomyolysis in patients with renal failure treated either with fibrates in the doses used in patients with normal GFR or treated simultaneously with statins have been published [7]. The patients with end-stage renal disease treated with hemodialysis may be particularly susceptible to toxic effects of fibrates since in the absence of the removal of their metabolites with urine only about 10% of a single fenofibrate dose was cleared from the blood by hemodialysis resulting in a substantially prolonged plasma half-life of its major metabolite fenofibric acid [15,16]. Since there have been no randomized trials in patients with renal failure treated with fibrates we decided to carry out a pilot placebo-controlled study with greatly reduced dose of fenofibrate in a group of chronic dialysis patients to assess the safety and lipid lowering efficacy of this drug. In addition, since some beneficial cardiovascular effects of fibrates that are potent PPAR-alfa agonists have been attributed to non-lipid lowering mechanisms [17] we decided to study the effect of fenofibrate on a biomarker of inflammation, i.e. high sensitive C-reactive protein and on plasma aminothiol redox status, i.e. a novel marker of oxidative stress [18].

## Patients and methods

In this double-blind randomized cross-over pilot single-center study 27 chronic Caucasian hemodialysis patients were included (18 men and 9 women, mean age 58.5 ± 13 years, time on dialysis 3.9 ± 4.0 years).

The patients were qualified if they fulfilled the following inclusion criteria: age >18 years, at least 12 months on chronic hemodialysis therapy with three dialysis sessions per week, total cholesterol concentration ≥200 mg/dL or LDL-cholesterol ≥120 mg/dL and serum triglycerides ≥150 mg/dL, residual diuresis <500 ml/day. The exclusion criteria were as follows: use of any fibrate or statin within 6 months prior to the study, previous intolerance of fibrates or statins, chronic immunosuppressive and/or steroid therapy, diabetes mellitus requiring insulin therapy, arterial hypertension resistant to treatment, cancer, acute or chronic inflammatory diseases, psychiatric disorders, history of poor compliance.

After being qualified the patients underwent a brief training aimed at explaining the study procedures, use of concomitant medications, compliance with pharmacotherapy and the necessity of reporting all the potential side-effects of the therapy at each dialysis to the treating physician.

At baseline visit in the morning of the day of a scheduled mid-week dialysis session the following parameters were measured in fasting patients: blood pressure with standard mercury sphygmomanometer (in triplicate), body height and body weight for body mass index (BMI) calculation, serum creatinine, urea, sodium, potassium, chloride, albumin, total, LDL and HDL cholesterol, triglycerides, phosphocreatine kinase, asparagine aminotransferase and C-reactive protein (high-sensitive assay). The blood was withdrawn immediately prior to dialysis after the vascular access cannulation. The HD prescription for all patients consisted of the treatment time of 240 min three times per week in a morning dialysis shift, blood flow of 200–250 ml/min and dialysate flow of 500 ml/min. In all cases low-flux modified cellulose (Hemophane) membranes were used (Alwall GFS Plus, Gambro, Lund, Sweden). Dialyzers were not reprocessed. Dialyzer surfaces were chosen according to the patient’s body surface area and the following sizes were used 1.1, 1.3 and 1.7 m^2^ resulting with ultrafiltration coefficients of 5.5, 6.8 and 9.4 ml/h/mmHg, respectively. Plasma total and reduced fractions of aminothiols (homocysteine, cysteine, cysteinylglycine and glutathione) were assessed with high-performance liquid chromatography (HPLC). Blood was withdrawn to the Sarstedt Monovette® system to prevent hemolysis. Separate prechilled EDTA-containing tubes were used for a collection of blood for the assessment of total and free aminothiol fractions. The blood for the measurement of total and total free aminothiol plasma concentration was immediately centrifuged in 4°C for 10 min (2 500 g) and the plasma was frozen until measurements [19] at −30°C for a maximum of 1 week. 

All other biochemical parameters were assessed with standard automated laboratory methods in the local laboratory.

**Figure 1:**
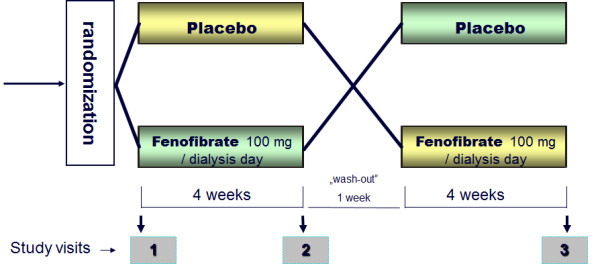
Diagram of study design.

All study procedures lasted 63 days. The above listed measurements were repeated three times, i.e. at baseline, after 28 days of treatment with fenofibrate or placebo and after 63 days when the patients completed the second treatment period. The patients were randomly assigned to receive placebo followed by fenofibrate or fenofibrate followed by placebo. Fenofibrate (or identically looking placebo tablets) was given in a single morning dose of 100 mg on a dialysis day shortly after an arrival to the center. The treatments were separated by a 7 day wash-out period. The study protocol is depicted in Figure 1.

The study protocol was approved by the Ethics Committee of the Medical University of Łódź, Poland.

All other medications that were chronically used by the patients were administered in unchanged doses for the whole duration of the study.

To compare the results of the measurements of plasma total and total free aminothiol fractions in hemodialysis patients to the reference values in subjects with normal renal function we used the results from the historic cohort of healthy volunteers (4 men and 4 women, mean age 38 ± 13 years) as characterized in our previous study [19]

### Statistical analysis

All results are expressed as mean ± SD. Statistical significance was defined at p < 0.05. The normality of data distribution was checked with Shapiro-Wilk test, and non-normally distributed data were logarithmically transformed before analysis. Within-group comparisons were made using a t-test or Wilcoxon’s test. The Pearson or Spearman correlation coefficient was used to assess relations between the variables. Statistical analysis of treatment outcome was carried out using the parametric approach to crossover trials including the evaluation of potential carryover effects on plasma lipids. Statistical analysis was performed using the StatSoft, Inc. Tulsa, OK, USA (2010). STATISTICA (data analysis software system), version 9.1. http://www.statsoft.com.

## Results

The treatment with the study drug was well-tolerated and no adverse effects of fenofibrate therapy were observed. All 27 patients that had been initially qualified completed the whole study.

No significant changes of blood pressure and body mass index were found throughout the study. There were also no significant changes of serum potassium, creatinine, urea and albumin and blood count with the exception of the leukocyte number that significantly decreased after fenofibrate but not after placebo when compared to baseline (6.4 ± 2.0 and 6.6 ± 2.0, respectively vs. 5,7 ± 1.8 x 10^9^; *p* < .01). There was a small but significant decrease of serum sodium after placebo but not after fenofibrate administration. Aspartate aminotransferase was within the normal range and remained unchanged for the whole study. The same was also observed in case of alanine aminotransferase. In contrast, creatine phosphokinase significantly increased during fenofibrate but not during placebo administration. Despite the significant increase of creatine phosphokinase its concentration remained in the normal range in each subject. Baseline and post-treatment results of blood pressure measurements and basic biochemical parameters are shown in Table 1.

**Table 1:** Blood pressure and basic biochemistry at baseline and after the 28-day treatment with fenofibrate or placebo

**Parameter**	**Baseline**	**After Placebo**	**After Fenofibrate**
Systolic blood pressure [mmHg]	138 ± 17	137 ± 12	134 ± 11
Diastolic blood pressure [mmHg]	91 ± 22	92 ± 18	93 ± 23
S-sodium [mmol/L]	139 ± 3	137 ± 3*	138 ± 3
S-potassium [mmol/L]	5.1 ± 0.9	5.1 ± 0.8	5.2 ± 0.8
S-albumin [g/L]	37.9 ± 3.4	38.4 ± 2.8	38.3 ± 3.3
S-urea [mg/dL]	128.2 ± 36.0	134.0 ± 28.1	139.1 ± 28.9
S-creatinine [mg/dl]	8.7 ± 2.4	8.8 ± 2.3	8.8 ± 2.3
S-GOT [IU/L]	21.1 ± 12.8	22.6 ± 9.1	23.3 ± 22.6
S-Creatine kinase [IU]	78.9 ± 48.8	72.9 ± 49.7	95.3 ± 63.9#
S-CRP [mg/L]	6.4 ± 5.2	7.2 ± 7.6	7.9 ± 9.1

# *p* = 0.01 vs baseline; * *p* < 0.05 vs baseline

Mean baseline CRP was increased but there were no changes in its concentration throughout the study.

**Figure 2:**
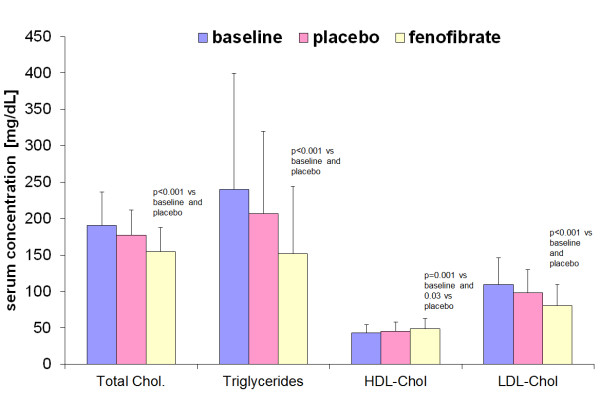
Plasma lipids at baseline and after the 28-day treatment with fenofibrate or placebo.

There were significant changes of all lipid fractions assessed in the study during the treatment with fenofibrate. Fenofibrate caused a significant decrease of total serum cholesterol (18.9% from the baseline valeu), LDL cholesterol (26%) and triglycerides (36.7%) and an increase of HDL cholesterol (12.1%). In contract placebo did not induce any significant changes of serum lipid concentration (Figure 2).

Table 2 shows plasma concentration of total aminothiols (glutathione, homocysteine, cysteine and cysteinylglycine). At baseline total plasma glutathione was lower in dialysis patients than in healthy controls but plasma homocysteine, cysteine and cysteinylglycine were all higher. 28-day treatment with fenofibrate caused a significant increase of total plasma glutathione (*p* = −.02) and homocysteine (p=,04) but not cysteine and cysteinylglycine.

**Table 2:** Plasma concentration of total aminothiols (glutathione, homocysteine, cysteine and cysteinylglycine) at baseline and after the 28-day treatment with fenofibrate or placebo

**Parameter**	Healthy controls*	**Baseline**	**After Placebo**	**After Fenofibrate**
Glutathione [nmol/mL]	11.9 ± 4.9	5.6 ± 1.4	5.6 ± 1.6	6.5 ± 1.7^#^
Homocysteine [nmol/mL]	11.3 ± 3.2	24.2 ± 12.0	26.9 ± 13.1	29.4 ± 20.2^##^
Cysteine [nmol/mL]	275 ± 99	535 ± 105	552 ± 107	542 ± 106
Cysteinylglycine [nmol/ml]	25 ± 7	58 ± 12	56 ± 10	56 ± 14

* healthy controls – historic cohort from Bald E et al. [19]

# *p* = 0.02 vs baseline; ## *p* = 0.04 vs baseline

Table 3 shows plasma concentration of total free thiols. Baseline plasma glutathione was lower and homocysteine, cysteine and cysteinylglycine higher in dialysis patients than in healthy controls. During the treatment with fenofibrate plasma free glutathione fraction increased significantly (*p* = .01) and free homocysteine decreased (*p* = .04). Both cysteine and cysteinylglycine were unchanged. Placebo did not have any effect on total free thiol fractions.

**Table 3:** Plasma concentration of total free aminothiols (glutathione, homocysteine, cysteine and cysteinylglycine) at baseline and after the 28-day treatment with fenofibrate or placebo

**Parameter**	Healthy controls*	**Baseline**	**After Placebo**	**After Fenofibrate**
Glutathione [nmol/mL]	5.3 ± 1.2	3.3 ± 0.9	3.4 ± 1.1	4.2 ± 1.4^#$^
Homocysteine [nmol/mL]	2.6 ± 1.7	6.6 ± 3.9	7.8 ± 5.0	9.0 ± 7.4^##^
Cysteine [nmol/mL]	47 ± 22	160 ± 48	163 ± 47	167 ± 51
Cysteinylglycine [nmol/ml]	7 ± 2	24 ± 4.5	23 ± 4	25 ± 7

* healthy controls – historic cohort from Bald E et al. [19]

# *p* = 0.01 vs baseline; $ *p* = 0.02 vs placebo

## *p* = 0.04 vs baseline

**Figure 3:**
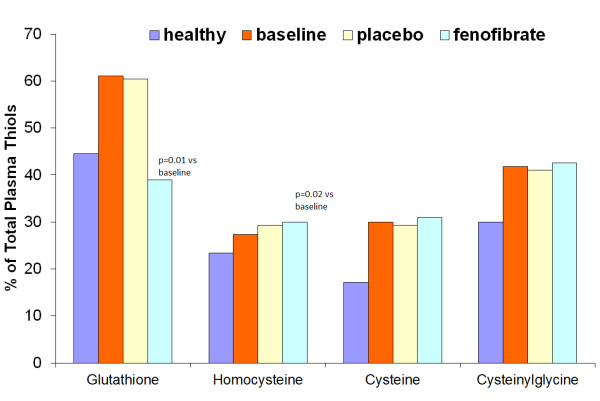
The ratio of free to total aminothiols (aminothiol redox status) at baseline and after the 28-day treatment with fenofibrate or placebo.

Figure 3 shows the ratio of free to total aminothiols (aminothiol redox status) during the study. The treatment with fenofibrate caused a significant decrease of the ratio of free to total glutathione (p = .01) and an increase in the ratio of free to total homocysteine (*p* = .02). No significant effect of placebo was seen in case of all aminothiols. The same was true during fenofibrate administration that did not induce any changes in the ratios of free to total cysteine and cysteinylglycine.

## **Discussion**

There are fundamental differences between the epidemiology of cardiovascular disease in end-stage renal disease and non-renal population [1,2]. Non-traditional risk factors may be responsible for a large part of cardiovascular mortality in chronic dialysis patients [1,2,20,21]. Furthermore the vascular pathologies commonly seen in dialysis patients may differ from typical atherosclerotic changes largely due to intensive medial and not only intimal calcification [22]. Both these processes may however share the same pathogenesis including chronic inflammation and oxidative stress [1,22]. C-reactive protein has been found to be a good biomarker of cardiovascular events and mortality both in the patient with and without renal disease [23]. There is also a good body of evidence that fenofibrate may effectively reduce C-reactive protein levels confirming therefore its anti-inflammatory effect [17,24], however the effect of this drug on cardiovascular endpoints, i.e. mortality is still a subject of controversy in particular after the publication of the final results of FIELD study [5] and a more recent Action to Control Cardiovascular Risk in Diabetes (ACCORD) trial [25,26]. In our study we were not able to show any significant effect of fenofibrate on a marker of inflammation, i.e. the serum C-reactive protein however there was a decrease in a number of leukocytes that is however much less sensitive marker of the chronic inflammatory process.

Reactive oxygen species may play an important role in the pathogenesis of renal failure and its complications since it was found that they are generated by most uremic toxins that possess strong redox properties [27]. This notion is corroborated by the finding of increased serum concentration of major aminothiols such as homocysteine and cysteine and decreased levels of glutathione in patients with chronic kidney disease when compared to subjects with normal renal function [28-30].

Both homocysteine and cysteine has been implicated in the pathogenesis of atherosclerosis through their effects on endothelial dysfunction and pro-thrombotic action [31,32]. Therefore homocysteine and cysteine are recognized as the biomarkers of cardiovascular disease [33,34]. Interestingly other aminothiols such as glutathione and cysteinylglycine were found to have the opposite effects since they have strong antioxidative and antiatherogenic action [35-37]. The most important role of thiol compounds *in vivo* is their function as redox buffers, regulating protein thiol-disulfide composition in both cellular and extracellular compartments [19]. Reduced, free oxidized and protein-bound forms of major thiols constitute the plasma thiol redox status [38]. In our study we measured both the total and total free fractions of aminothiols to avoid a direct measurement of the reduced forms which are highly unstable and therefore the methods of their measurement cannot be standardized [19,38]. Therefore as was postulated almost two decades ago by Ueland et al. [38] the ratio of total free to total aminothiol may reflect a redox thiol status, i.e. an activity of the major extracellular antioxidant defense system. Since in our study the treatment with fenofibrate shifted an aminothiol balance towards a less prooxidative pattern it may indirectly show the protective effect of this agent on cardiovascular system however this concept will require further confirmation with alternative methods of oxidative stress measurement.

It has been well proven that dyslipidemia is much more common in patients with end-stage renal disease than in subjects with normal or only mildly impaired renal function [10,39,40]. The dyslipidemic pattern of uremic dyslipidemia that is characterized by increased triglyceride and LDL-cholesterol and decreased HDL cholesterol makes dialysis patients almost ideal candidates for treatment with fibric acid derivatives [10,41]. Unfortunately, most fibrates with the exception of probucol are excreted with urine and may accumulate in particular in patients with advanced and end-stage chronic kidney disease [15,16,42,43]. The fibrates are also poorly removed by hemodialysis [15]. Therefore the evidence of the effects on fibrates in chronic kidney disease from large, randomized trials is missing [41,42] with the exception of a recent post-hoc analysis from FIELD study that confirmed the safety and potent lipid-lowering efficacy of fenofibrate but that observation was limited only to the patients with mildly to moderately impaired kidney function [44]. In contrast, statins are less accumulated in case of renal function impairment and most of them can be safely used even in dialysis patients after a dose reduction [45]. To avoid any potential toxicity in this pilot study we decided to reduce a dose of fenofibrate with its administration limited to dialysis days. This also secured a good compliance since the patients received the drug only in the dialysis center upon their arrival for the scheduled dialysis treatment.

In our study no side-effects of fenofibrate were observed and in particular none of our patients report any such complaints as muscle weakness that could have indicated skeletal muscle toxicity. That might be also due to the fact that we excluded the subjects that were simultaneously using statins as the risk of rhabdomyolysis is the greatest when fibrate and statin are used in combination [7,8]. We observed small but significant increase of creatine kinase. It is however of note that despite the increase of serum creatinine kinase its serum levels remained within normal range. Furthermore another common serum marker of skeletal muscle toxicity of fibrate, i.e. asparagine aminotransferase was unchanged during the treatment.

Although our study was carefully designed, controlled and randomized with a cross-over comparison of a study medication and placebo it was too small and too short to investigate any hard-end points. Therefore we were able to focus only on the surrogate biochemical markers of inflammation and oxidative stress. Other limitations of our design are as follows: it was carried out in a single dialysis center and all the patients were Caucasian, there was no subgroup analysis based on etiology of kidney failure. Furthermore no pharmacokinetic study with different doses of study was performed with the measurement of plasma levels of fenofibrate and its active metabolites. Although only the pharmacokinetic data could provide the information that the patients were fully compliant with the treatment any non-adherence is highly unlikely in case of our study because the dosing scheme comprised the administration of a study drug only on dialysis days under a supervision of a staff at the dialysis unit.

In summary we found that low-dose fenofibrate therapy is an effective treatment of uremic dyslipidemia. Although the safety profile of fenofibrate has been satisfactory in this small trial in chronic hemodialysis patients our findings will require confirmation in a larger and longer study since the potential toxicity of fenofibrate may develop over a much longer time of treatment. Such studies that may lead to more effective treatment of lipid disorders in end-stage kidney disease would be very important for our futire clinical practice since hyperlipidemic patients with end-stage chronic kidney on long-term dialysis are very difficult to treat due to frequent side-effects of the drugs and advanced atherosclerosis due to long-standing uremic milieu. That was probably the reason why even the statin therapy failed to show any beneficial effect on cardiovascular events and mortality in this population in most large trials such as 4D and Aurora with the recent exception of the largest SHARP study [46].

## Competing interests

The authors declare no conflict of interest. The study was supported by the Medical University of Lodz grant No 503/5-139-01/503-01.

## Authors’ contributions

AM and PD conducted the clinical part of the study, MN, EB and GC designed the study, AM, MN and EB analyzed the data and wrote the manucript, GC carried out laboratory measurements and analyzed their results. All authors read and approved the final manuscript.
